# Toll-like receptor (TLR)-mediated innate immune responses in the control of hepatitis B virus (HBV) infection

**DOI:** 10.1007/s00430-014-0370-1

**Published:** 2014-12-31

**Authors:** Ejuan Zhang, Mengji Lu

**Affiliations:** Institute of Virology, University Hospital Essen, University of Duisburg-Essen, Hufelandstrasse 55, 45122 Essen, Germany

**Keywords:** Toll-like receptor (TLR), Hepatitis B virus (HBV), Chronic viral infection, Interferon (IFN), Pro-inflammatory cytokines, Innate immunity

## Abstract

The role of adaptive immune responses in the control of hepatitis B virus (HBV) infection is well accepted. The contribution of innate immune responses to the viral control is recognized but yet not fully understood. Toll-like receptors (TLRs) sense pathogen-associated molecule patterns and activate antiviral mechanisms including the intracellular antiviral pathways and the production of antiviral effectors like interferons (IFNs) and pro-inflammatory cytokines. Activation of the TLR3 pathway and the production of IFN-β represent one of the major mechanisms leading to the suppression of HBV replication in the liver, as shown in different in vitro and in vivo models. TLR4 signaling and TLR2 signaling result in the activation of intracellular pathways including MAPK and PI-3 K/Akt in hepatocytes and reduce HBV replication in an IFN-independent manner. HBV is able to counteract the actions of TLR3 and TLR2/4 through downregulation of TLR expression and attenuation of the cellular signaling pathways. Thus, TLR ligands are promising candidates as immunomodulators and therapeutics for the treatment of chronic HBV infection. Specific antiviral treatment against HBV could recover the TLR functions in chronic HBV infection and increase the effectiveness of therapeutic approaches based on TLR activation.

## Introduction

According to the estimation of World Health Organisation, there are about 240 million people who are chronically infected with hepatitis B virus (HBV). The chronic HBV infection is one of the major causes of hepatocellular carcinoma and liver cirrhosis. There is a large body of evidence for the essential role of cell-mediated immune responses for viral clearance in acute HBV infection. Patients with chronic HBV infection fail to develop adequate HBV-specific immune responses [1]. Standard treatment regimens with pegylated interferon (IFN)-*α* and nucleoside/nucleotide analogs are used for therapy of chronic hepatitis B but are only partially successful. Several recent studies indicated the possibility of stimulating specific immune responses against HBV in chronically infected patients. The recent approaches were summarized in previous reviews of our group and others and in the present issue [2–5]. A number of therapeutic vaccination trials using conventional HBV vaccines failed to demonstrate the effectiveness in terms of the induction of HBV-specific immune responses and suppression of HBV replication in chronic HBV carriers [4, 5]. New approaches based on DNA vaccines or anti-HBs antibody-HBs immune complex are now being tested in clinical trials [6–8]. As a principle recognized on the basis of available information, a combined strategy including antiviral treatment and immunomodulation will be needed to stimulate the full range of immune responses to achieve effective control over HBV infection. Important aspects of the human HBV infection have been studied with a genetically closely related virus of *Hepadnaviridae*, woodchuck hepatitis virus (WHV), which infects a North American rodent, the woodchuck. In the woodchuck model, combinations of antiviral treatment and therapeutic vaccinations led to the induction of specific T cell and B cell responses to WHV antigens and sustained suppression of WHV replication in some individual animals [2, 4, 9, 10]. Stimulation of innate immune responses may further improve the immunotherapeutic effect of combination strategies against the hepadnaviral infection.

### Toll-like receptor (TLR) system

**Fig. 1:**
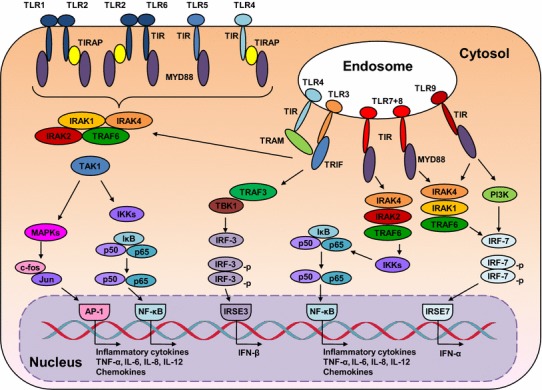
TLR signaling. Upon the activation of TLRs by their respective ligands, the adaptor molecules MYD88, TIRAP, TRIF, and TRAM are recruited and further activate the kinases TAK1, MAPKs, TRAF3, TBK1, and IKKs, resulting in nuclear translocation of transcriptions factors AP-1, NF-κB, IRF3, or IRF7, and subsequent transcription of IFNs and pro-inflammatory cytokines

The significance of the innate immune response as a defense against microbial infections and its link to the adaptive immune responses has been recognized during the past years. Toll-like receptors (TLRs) are a group of highly conserved molecules that play a critical role in the recognition of pathogen-associated molecular patterns (PAMPs) and in the activation of innate immune responses to infectious agents [11]. TLRs are structurally characterized by an ectodomain composed of leucine-rich repeats for binding and recognition of PAMPs and a cytoplasmic domain homologous to the cytoplasmic region of the interleukin (IL)-1 receptor, known as the TIR domain, for downstream signaling [12]. TLR ligands are natural macromolecular components derived from pathogens and may be composed of lipids, lipopeptides, proteins, and nucleic acids. Some synthetic small molecules could mimic TLR ligands and activate TLR-mediated cellular signaling. A subgroup within the TLR family including TLR3, TLR7, TLR8, and TLR9 is localized in endosomes and recognizes nucleic acids such as viral DNA or RNA. The other subgroup of surface-expressed TLR1, TLR2, TLR4, TLR5, and TLR6 recognizes extracellular bacterial and fungal cell wall components, as well as some viral proteins [13, 14]. Binding of TLR agonists to their receptors initiates the activation of complex networks of intracellular signal transduction pathways to coordinate the inflammatory response. Conformational changes and dimerization of TLRs occur upon binding with ligands. The important components of these signaling networks are the adaptor proteins and several protein kinases including ERK, JNK, p38 MAP kinase, and PI-3 k kinase, and the transcription factors IRF3/5/7, nuclear factor kappa B (NF-κB), and AP-1. The activation of these transcription factors leads to the induction of type I IFNs, pro-inflammatory cytokines, or co-stimulatory molecules, which are involved in antiviral responses [15, 16]. The crucial adaptor proteins including myeloid differentiation primary-response protein 88 (MyD88), used by nearly all TLRs except TLR3, TIR domain-containing adaptor protein (TIRAP), TIR domain-containing adaptor protein inducing interferon (IFN)-β (TRIF), and TRIF-related adaptor molecule (TRAM) are recruited [17]. TLR4 is unique among TLRs being able to activate two distinct signaling pathways, TIRAP/MyD88 and TRAM/TRIF [17]. The MyD88-dependent pathway leads to the activation of downstream signal transduction involving IL-1R-associated kinases (IRAKs), tumor necrosis factor receptor (TNFR)-associated factor 6 (TRAF6), transforming growth factor (TGF)-β-activated kinase (TAK1), and the inhibitor of nuclear factor-κB (IκB) kinase complex. Through the NF-κB and activating protein 1 (AP1), the MyD88-dependent TLR activation results in the production of pro-inflammatory cytokines IL-6, IL-10, IL-12, and TNF-α. In contrast, the TRIF-dependent pathway leads to the activation of IFN regulatory factors (IRFs) and production of type I IFNs [15, 17]. Exceptionally, plasmacytoid dendritic cells produce type I IFN after TLR7 and TLR9 activation via the MyD88-IRF7-dependent pathway [18]. Figure 1 schematically depicts human TLR signaling pathways.

### Recognition of HBV by host cells

There is accumulating evidence that the innate branch of the host immune system plays an important role in the control of HBV infection [19–21]. The previous studies in chimpanzees and in patients showed that HBV infection does not lead to a measurable response involving type I IFNs. Wieland et al. [22] investigated the transcriptome of the liver in three chimpanzees during the course of acute HBV infection. Their analysis focused on two diverse groups of cellular genes: Those in the early phase are associated with the innate immune response, and those in the late phase are associated with the adaptive immune response that terminates infection. They demonstrated that HBV does not induce any genes during entry and expansion, leading the authors to suggest that HBV is a “stealth virus” in the early phase of infection. By contrast, a large number of IFN-γ-regulated genes are expressed in the liver during viral clearance. The upregulation of IFN-γ-regulated genes in the liver results from the adaptive T cell response as specific T cells infiltrating the liver are major producers of IFN-γ [22]. Thus, HBV infection strongly differs from other viral infections like HCV infection in the early phase, as HCV induces a strong IFN-α response in chimpanzees [23]. Dunn et al. [24] measured type I IFN production in patients with acute HBV infection and found only a low level of type I IFN not higher than those found in healthy controls. Similarly, IL-15 and IFN-λ1 were not induced during peak viraemia. In contrast, IL-10 is induced at the early stages of acute HBV. The lack of early IFN response in vivo during acute HBV infection does not necessarily exclude the triggering of host IFN responses by HBV. HBV infection may be initiated only with few viral particles and may not induce a host response at the initial phase of infection that is measurable by gene array technology or cytokine detection. In addition, HBV may inhibit host IFN responses by specific mechanisms as described below. Experimental data are available, indicating that HBV interacts with the host innate immune system but is able to inhibit host responses. It has been shown that HBV interacts with hepatic non-parenchymal cells (NPCs) and induces the production of IL-6 [25], though it is not clear how hepatic NPCs sense HBV. Within 3 h, these cells release inflammatory cytokines including IL-1β, IL-6, IL-8, and TNF-α without inducing an IFN response. IL-6 is able in turn to inhibit the expression of hepatocyte nuclear factor (HNF) 1α and HNF 4α, two transcription factors essential for HBV gene expression and replication. However, relatively high doses of HBV particles are usually used in such experiments to induce measurable responses of host cells. Future in vivo experiments are required to compare and verify these findings.

### TLR3 and HBV

TLR3 activation results in the production of type I IFNs in different cell types. IFN-β has been identified as a major antiviral factor produced by NPCs in response to TLR3 [26]. Wieland et al. [27] showed that TLR3 ligand poly I:C induces intrahepatic IFN-β production and inhibits HBV replication by non-cytolytic mechanisms that either destabilize pregenomic (pg)RNA-containing capsids or prevent their assembly. Isogawa et al. [28] tested the ability of different TLR ligands to inhibit HBV replication in the HBV transgenic mouse model. Consistently, a single-dose injection of TLR3, TLR4, TLR5, TLR7, and TLR9 ligands suppressed HBV replication in the liver in an IFN-α/β-dependent manner. In a doxycycline (dox)-inducible HBV replication system, IFN-β pretreatment prevents the production of replication-competent pgRNA-containing capsids but does not change the turnover rate of preformed HBV RNA-containing capsids [29]. Apparently, the formation of replication-competent HBV capsids is one of the major targets of IFN-mediated antiviral actions. A great number of cellular IFN-stimulated genes (ISGs) are activated by IFNs and may inhibit the different steps of the HBV life cycle [30]. A recent publication suggests that HBV covalently closed circular (ccc) DNA could be degraded by the action of APOBEC3A and 3B cytidine deaminases [31]. These findings partly explain the therapeutic effect of IFN-α in patients with chronic HBV infection. IFN-α is widely used to treat chronic HBV infection and can lead to sustained decrease of HBsAg and virus clearance in about 30 % of chronically HBV-infected patients. In addition, type I IFNs may modulate specific immune responses to HBV, resulting in HBe seroconversion or complete control of HBV infection.

TLR3 activation of hepatic NPCs could lead to IFN-β production and HBV inhibition in in vitro [26, 32, 33]. Upon poly I:C stimulation, hepatic NPCs such as Kupffer cells (KC) and liver sinusoid endothelial cells (LSEC) release antiviral cytokines which are able to inhibit HBV replication in a co-culture model utilizing HBV-Met cells that contains an integrated HBV genome [34]. Blocking with specific antibodies to type I and II IFNs identified IFN-β as the major anti-HBV factor produced by poly I:C-treated NPCs [26]. While HBV DNA replicative intermediates were efficiently suppressed, viral mRNAs as well as secretion of HBsAg and HBeAg remained largely unchanged. Importantly, screening of different TLR ligands demonstrated that hepatic NPCs show a significant production of IFN-β only in response to TLR3 stimulation (and a lower extent to TLR4 stimulation, see below). Therefore, TLR3-mediated response and IFN-β production in the liver may contribute to the control of pathogens including HBV in a unique way.

Several studies suggest that HBV is able to inhibit pattern recognition receptor (PRR) and IFN signaling. HBV surface and “e” antigen (HBsAg, HBeAg) and HBV particles could inhibit the activation of NPCs by TLR3 ligands [35]. Co-culture of hepatic NPCs with HBV-Met cell supernatants, HBsAg, HBeAg as well as HBV virions results in abrogation of TLR-induced antiviral activity, correlating with decreased activation of IRF3, NF-κB, and ERK1/2 in NPCs. Our most recent data suggest that HBsAg may trigger IL10 production on hepatic cells and thereby attenuates the TLR3-mediated activation of NPCs [33]. This is consistent with the recent publication that HBsAg induces TNF-α and IL-10 production by monocytes which leads to downregulation of TLR9 expression on pDCs, thereby inhibiting IFN-α production by pDCs [36]. At high amounts of HBV, even TLR-induced expression of TNF-α and IL-6 in NPCs was suppressed. In addition, many studies provide evidence that HBV polymerase may counteract the innate responses at two or more steps: (1) HBV polymerase is able to inhibit the IRF3 activation by interacting with RNA helicase DDX3 in hepatoma cells [37, 38]; (2) HBV polymerase is able to block IFN signaling by inhibition of PKC-δ-mediated phosphorylation of stat 1 and importin-dependent translocation of stat 1/stat 2/IRF9 complex [39, 40]. HBx protein was reported to promote the decay of mitochondrial antiviral signaling protein (MAVS), the adaptor of RIG-I, and MDA5 receptors [41, 42]. However, the relevance of these findings for the human HBV infection remains to be defined.

**Fig. 2:**
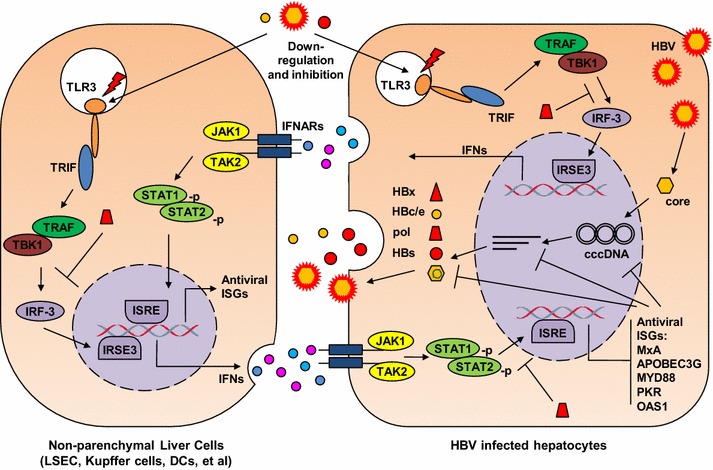
Interaction of HBV and TLR3. TLR3 activation in hepatic NPCs leads to the production of IFN-β and subsequently the upregulation of ISGs in hepatocytes. Antiviral ISGs like MxA and IFIT1/2 inhibit HBV replication at the transcriptional and posttranscriptional steps. HBV virions and proteins are able to suppress TLR3 signaling and block IFN-β production. HBV polymerase could interfere with IRF3 action and block the nuclear translocation of Stat1/2

TLR3-mediated functions are impaired in patients with chronic HBV infection and may recover partially under successful antiviral treatment [43]. In the woodchuck model, PBMCs from animals with chronic WHV infection show reduced responses to poly I:C stimulation [44]. Taken together, the interaction of HBV or the molecular components of HBV with the innate immune system is complex, leading both to activation and inhibition of host innate responses. Figure 2 depicts the interaction of TLR3 signaling pathway with HBV in a schematic way.

### TLR2/4 and HBV

Unlike to TLR3, TLR4 activation by lipopolysaccharide (LPS) leads to low IFN-β production only in KCs but not in LSECs and hepatocytes [26]. However, TLR4-activated KCs release other yet undefined antiviral factors, inhibiting HBV replication in HBV-Met cells. In contrast to TLR3 ligands, Zhang et al. [45] demonstrated that activation of cellular pathways by TLR4 ligands leads to inhibition of hepadnaviral replication. In the model of WHV-infected primary hepatocytes (PWHs), LPS stimulation led to a pronounced reduction of WHV replication intermediates without a significant IFN induction, while poly I:C transfection resulted in the IFN production and a highly increased expression of antiviral genes in PWHs, but only slight inhibitory effect on WHV replication. LPS was able to activate NF-κB, MAPK, and PI-3 k/Akt pathways in PWHs. The inhibitors of MAPK-ERK and PI-3 k/Akt pathways, but not those of IFN signaling pathways, block the antiviral effect of LPS, indicating that IFN-independent pathways which activated by LPS are able to downregulate hepadnaviral replication in hepatocytes [45].

**Fig. 3:**
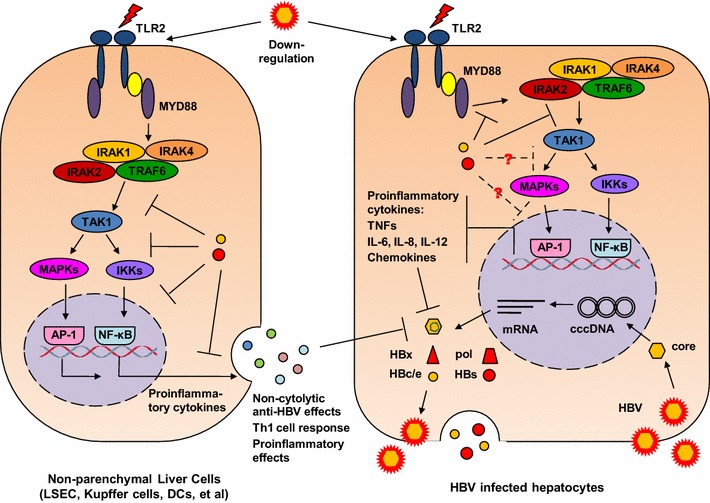
Interaction of HBV and TLR2/4. TLR2/4 activation in hepatocytes inhibits HBV replication in an IFN-independent manner but requires the participation of intracellular signaling pathways like MAPK pathway. TLR4 stimulation in KCs leads to the production of IFN-β and an unknown antiviral effector which inhibits HBV replication in hepatocytes. HBV downregulates TLR2/4 expression during chronic HBV infection. HBsAg and HBeAg are able to block TLR2 signaling at different steps, preventing the production of pro-inflammatory cytokines

TLR2 and TLR4 share the cellular MyD88-dependent signaling pathway in mammalian cells (Fig. 3). Consequently, TLR2 and TLR4 mediate the activation of the same signaling pathways downstream of MyD88, including NF-κB, MAPK, and PI-3 k/Akt pathways. Similarly, TLR2 is able to inhibit HBV or WHV replication in human hepatoma cells or PWHs [46, 47]. Again, the antiviral action of TLR2 is dependent on the presence of adaptor molecules like TAK1, IRAK1/4, and TRAF6 and the downstream MAPK and PI-3 k/Akt pathways [47]. Silencing of the expression of adaptor molecules or blocking the MAPK and PI-3 k/Akt pathways with chemical inhibitors significantly enhanced HBV replication. In the HBV transgenic mouse model, the injection of a single dose of TLR2 ligands reduced HBV replication in the liver but not as effective as IFN-inducing ligands [28]. It was not examined whether TLR2 ligands also activate MAPK and PI-3 k/Akt pathways in vivo and thereby exert the antiviral action.

TLR2 activation and TLR4 activation lead to the production of pro-inflammatory cytokines IL6 and TNF-α in hepatic NPCs and hepatocytes [32, 45, 47, 48]. Though the antiviral effect of TLR2 and TLR4 ligands does not directly depend on the production of pro-inflammatory cytokines, IL6 and TNF-α have been shown to inhibit HBV replication in primary hepatocytes [25, 49]. Xu et al. [49] explored the Tupaia model to investigate the effect of TNF-α on HBV infection. Stimulation of HBV-infected primary Tupaia hepatocytes with recombinant Tupaia TNF-α led to viral suppression, while covalently closed circular DNA and viral RNA were still detectable, leading to the conclusion that TNF-α may also contribute to control HBV infection.

Obviously, HBV developed measures to counteract the antiviral functions mediated by TLR2. Using hepatocytes and KCs isolated from liver biopsies of patients with CHB, Visvanathan et al. [50] showed significantly decreased TLR2 expression on hepatocytes, KCs, and peripheral monocytes in patients with HBeAg-positive CHB in comparison with HBeAg-negative CHB and controls. The level of TLR4 expression did not significantly differ between these groups. Hepatic cell lines harboring a recombinant baculovirus encoding HBV significantly reduced TNF-α expression as well as phospho-p38 kinase expression in the presence of HBeAg. In the absence of HBeAg, HBV replication was associated with upregulation of the TLR2 pathway resulting in increased TNF-α expression [50]. HBeAg was found to co-localize with Toll/IL-1 receptor (TIR)-containing proteins TRAM, Mal, and TLR2, interact with TIR proteins Mal and TRAM, and disrupt the homotypic TIR–TIR interaction. Consequently, HBeAg suppressed TIR-mediated activation of the inflammatory transcription factors, NF-κB, and interferon-β promoter activity [51]. Consistently, TLR2 expression was found to be significantly suppressed in PBMCs from chronically HBV-infected patients and in woodchuck liver tissue and PBMCs if chronically infected with WHV [47, 52]. Previously, Wu et al. [39] demonstrated that HBV blocks the MyD88 expression, the central adaptor molecule in TLR-mediated innate immune responses, by an antagonistic activity of the terminal protein (TP) domain of the HBV polymerase. It could be shown that HBV polymerase is able to block the nuclear translocation of stat 1 thus representing a general inhibitor of IFN signaling [40] and IFN-inducible MyD88 expression.

Activation of innate immunity is a prerequisite for proper adaptive immune responses. As an example, TLR2 is expressed widely such on antigen-presenting cells (APC), endothelial and epithelial cells as well as on T-lymphocytes on which it acts as a costimulatory molecule. Wu et al. [32] found previously that TLR2 ligands could trigger the expression of costimulatory molecules on hepatic NPCs. LSECs are unique organ-resident antigen-presenting cells capable of antigen cross-presentation and reported to prime naïve CD8+ T cells to memory cells at non-inflammatory conditions [53]. Under certain conditions, LSECs could also directly promote T cell immunity [54]. Recently, we examined functional maturation of LSECs by TLR ligand stimulation, demonstrating that pretreatment of LSECs with TLR1/2 ligand but not TLR3 and TLR4 ligands reverts their suppressive properties to induce specific T cell immunity [55]. IL-12 was identified to be one essential mediator for LSEC-mediated CD8+ T cell immunity, which was produced at a low level but sustainably after TLR2 stimulation. Our findings suggest that TLR2 activation has a great impact on T cell immunity in the liver and may be used to stimulate specific immune responses to persistent infection of HBV and HCV.

On the other hand, Wang et al. [56] could show that HBsAg inhibits TLR2-mediated stimulation of human PBMCs and IL12 production. In the presence of HBsAg, both Pam3CSK4-triggered IL-12p40 mRNA expression and IL-12 production in phorbol 12-myristate 13-acetate (PMA)-differentiated THP-1 macrophages are reduced in a dose-dependent manner, while the production of IL-1β, IL-6, IL-8, IL-10, and TNF-α is not affected. The presence of HBsAg inhibits the TLR2-mediated activation of NF-κB and MAPK signaling, by selective impairment of JNK-1/2 and c-Jun phosphorylation. Thus, HBsAg likely interferes with the initiation of adaptive immune responses by selective inhibition of TLR2-stimulated IL-12 production in monocytes.

### Therapeutic approaches

The findings mentioned above suggest that TLR ligands may be used for therapeutic approaches against chronic viral infection. However, only few trials using TLR ligands for therapies against viral infections have been carried out until now [57]. CpG oligonucleotides (ODN), ligands of TLR9, have been considered as promising candidates. A large number of new reagents that are potentially suitable as immunomodulators and therapeutics the sequence could be generated by modifications of CpG ODN [58, 59]. However, a candidate CpG ODN has been tested in clinical trials and the woodchuck model for treatment of chronic hepatitis C and B but failed to show sufficient therapeutic effect if applied alone. TLR3 and TLR4 ligands are not tested in clinical trials yet. Recently, TLR7 ligand GS-9620 has been examined for its antiviral effect in the woodchuck and chimpanzee models. Interestingly, a 4-week treatment with GS-9620 resulted in a sustained, marked reduction of serum WHV DNA and WHV surface antigen (WHsAg) levels and in the induction of anti-WHs antibody response, as well as a markedly decreased incidence of hepatocellular carcinoma in chronic WHV-infected woodchucks [60]. In chimpanzees, GS-9620 induced an increase of serum IFN-α in a dose-dependent manner and triggered the ISG expression in PBMCs and the liver. A reduction of HBV viral load and serum HBsAg was observed in three chronically HBV-infected chimpanzees treated with GS-9620 [61]. TLR7/8 ligands are promising drug candidates if their toxicity could be reduced to a tolerable range.

A potential use of TLR ligands as adjuvants for therapeutic vaccines has been considered for long time. The rational design of specific TLR agonists may increase potency and tolerability of new adjuvants and provides the opportunity to meet the stringent safety criteria for new vaccine formulation [62]. Two improved adult HBV vaccines Fendrix and Supervax using TLR4 agonists as adjuvant are now on the market [63, 64]. Monophosphoryl lipid A (MPLA), a chemically modified derivative of the lipid A moiety of LPS, is considerably less toxic but has similar immunostimulatory activity [65]. MPLA-formulated hepatitis B vaccines elicit protective anti-HBs antibody titers with only two injections instead of three [64]. A class B CpG ODN called 1018 ISS in combination with recombinant HBsAg has been tested in a Phase III clinical trial for persons older than 40 years of age. This vaccine formulation increases seroprotection rates to 100 %, compared with a rate of only 64 % in the alum-adjuvanted rHBsAg group [66]. Such a vaccine formulation may be used for patients with impaired immune system, as it is more effective in the hypo-responsive population than conventional HBV vaccines [67].

### Perspectives

Based on the current knowledge, TLR-mediated innate responses may not control HBV infection alone. Though stimulation with TLR ligands reduces HBV replication in hepatocytes, the TLR-mediated antiviral action against HBV is far less efficient than those achieved by nucleoside analogs. The innate immune system is known to respond fast and aimed to slow down viral spread in primary infections; thus, the link from innate to adaptive immune responses may be more important for the control of viral infection. The innate immune system plays a pivotal role for the regulation of adaptive immunity [68]. Recently, it was shown that poly I:C treatment leads to HBV clearance in hydrodynamic injection mouse model [69]. In this model, HBV clearance required IFN-α and IFN-γ, indicating a complex mode of poly I:C-triggered action. CXCR3 was also essential for HBV clearance after poly I:C injection, apparently responsible for the recruitment of T cells. Other studies conducted by several groups have shown that TLR2 is expressed on activated and memory CD4+ and CD8+ T cells and serves as co-stimulatory molecule to enhance their proliferation, survival, and functions [70–73]. TLR2 agonists stimulate activated T cells thus promoting their proliferation and differentiation in vitro and in vivo. Consistently, TLR2 ligand Pam3CSK4 application in vivo with transferred tumor antigen (Ag)-specific CD8+ T cells results in enhanced therapeutic efficacy of these CD8+ T cells in tumor models [74, 75]. Moreover, covalent linkage of TLR2 ligand Pam3CSK4 or Pam2CSK4 with peptides representing CD8+ T cell or B cell epitopes efficiently primed respective specific CD8+ T cell or B cell immune responses in vivo [76–79]. It was shown that TLR2 engagement on CD8+ T cells increased T-bet transcription in a MyD88-Akt-mTOR- and protein kinase C-dependent manner [71]. The molecular mechanisms underlying TLR2-mediated T cell proliferation and functional differentiation in HBV infection need in-depth analysis. Thus, future research should not only investigate the direct antiviral effect of TLR-mediated action but also analyze and optimize the connection of innate and adaptive immune responses. In the specific case of TLR2, it is desirable to identify specific markers expressed on CD8+ T cells after TLR2 stimulation. Such markers may facilitate future analysis of CD8+ T cells in vitro and vivo and understanding the inhibitory action of HBV on TLR2 co-stimulation of CD8+ T cells. These approaches could also be extended to studies about other TLRs.
